# Large-scale analysis of full-length cDNAs from the tomato (*Solanum lycopersicum*) cultivar Micro-Tom, a reference system for the Solanaceae genomics

**DOI:** 10.1186/1471-2164-11-210

**Published:** 2010-03-30

**Authors:** Koh Aoki, Kentaro Yano, Ayako Suzuki, Shingo Kawamura, Nozomu Sakurai, Kunihiro Suda, Atsushi Kurabayashi, Tatsuya Suzuki, Taneaki Tsugane, Manabu Watanabe, Kazuhide Ooga, Maiko Torii, Takanori Narita, Tadasu Shin-i, Yuji Kohara, Naoki Yamamoto, Hideki Takahashi, Yuichiro Watanabe, Mayumi Egusa, Motoichiro Kodama, Yuki Ichinose, Mari Kikuchi, Sumire Fukushima, Akiko Okabe, Tsutomu Arie, Yuko Sato, Katsumi Yazawa, Shinobu Satoh, Toshikazu Omura, Hiroshi Ezura, Daisuke Shibata

**Affiliations:** 1 Kazusa DNA Research Institute, 2-6-7 Kazusa-Kamatari, Kisarazu, 292-0818, Japan; 2 Meiji University, 1-1-1 Higashi-mita, Tama-ku, Kawasaki, 214-8571, Japan; 3 Chiba Prefectural Agriculture and Forestry Research Center, 808 Daizenno-cho, Midori-ku, Chiba, 266-0006, Japan; 4 National Institute of Genetics, Yata 1111, Mishima, 411-8540, Japan; 5 Tohoku University, 1-1 Amamiya-machi, Tsutsumidori, Aoba-ku, Sendai, 981-8555, Japan; 6 The University of Tokyo, Komaba, Meguro-ku, 153-8902, Japan; 7 Tottori University, 4-101 Koyama-minami, Tottori, 680-8553, Japan; 8 Okayama University, 1-1-1 Tsushima-naka, Kita-ku, Okayama, 700-8530, Japan; 9 Tokyo University of Agriculture and Technology, 3-5-8 Saiwai-cho, Fuchu, 183-8509, Japan; 10 Institute of Biological Sciences, University of Tsukuba, 1-1-1 Tennodai, Tsukuba, 305-8571, Japan; 11 Gene Research Center, University of Tsukuba, 1-1-1 Tennodai, Tsukuba, 305-8571, Japan

## Abstract

**Background:**

The Solanaceae family includes several economically important vegetable crops. The tomato (*Solanum lycopersicum*) is regarded as a model plant of the Solanaceae family. Recently, a number of tomato resources have been developed in parallel with the ongoing tomato genome sequencing project. In particular, a miniature cultivar, Micro-Tom, is regarded as a model system in tomato genomics, and a number of genomics resources in the Micro-Tom-background, such as ESTs and mutagenized lines, have been established by an international alliance.

**Results:**

To accelerate the progress in tomato genomics, we developed a collection of fully-sequenced 13,227 Micro-Tom full-length cDNAs. By checking redundant sequences, coding sequences, and chimeric sequences, a set of 11,502 non-redundant full-length cDNAs (nrFLcDNAs) was generated. Analysis of untranslated regions demonstrated that tomato has longer 5'- and 3'-untranslated regions than most other plants but rice. Classification of functions of proteins predicted from the coding sequences demonstrated that nrFLcDNAs covered a broad range of functions. A comparison of nrFLcDNAs with genes of sixteen plants facilitated the identification of tomato genes that are not found in other plants, most of which did not have known protein domains. Mapping of the nrFLcDNAs onto currently available tomato genome sequences facilitated prediction of exon-intron structure. Introns of tomato genes were longer than those of Arabidopsis and rice. According to a comparison of exon sequences between the nrFLcDNAs and the tomato genome sequences, the frequency of nucleotide mismatch in exons between Micro-Tom and the genome-sequencing cultivar (Heinz 1706) was estimated to be 0.061%.

**Conclusion:**

The collection of Micro-Tom nrFLcDNAs generated in this study will serve as a valuable genomic tool for plant biologists to bridge the gap between basic and applied studies. The nrFLcDNA sequences will help annotation of the tomato whole-genome sequence and aid in tomato functional genomics and molecular breeding. Full-length cDNA sequences and their annotations are provided in the database KaFTom http://www.pgb.kazusa.or.jp/kaftom/ via the website of the National Bioresource Project Tomato http://tomato.nbrp.jp.

## Background

The Solanaceae family comprises 1000-2000 species that show wide morphological variability and ecological adaptability [1]. This taxon includes a number of vegetable crops including fruit-bearing vegetables, tuber-bearing vegetables, and ornamental plants, many of which have economic importance. Tomato (*Solanum lycopersicum*) is a member of the Solanaceae, and it has served as a model system for fruit development [2] and plant defense [3,4]. A number of studies have accumulated substantial information regarding the genetics and physiology of tomato.

To understand the physiological processes of tomato at the molecular level, it is necessary to link traits to DNA sequence. Whole-genome sequencing is one of the approaches to achieve this goal. A project for sequencing the tomato genome was launched by The International Solanaceae Project (SOL) in the year 2003, and the project is currently in the final stage of sequence polishing (March, 2010) [4]. Accumulation of expressed sequence tags (ESTs) is an alternative approach to collecting DNA sequence information. A large number of ESTs have been produced to provide a resource for gene finding and expression studies [5-9]. For tomato ESTs, several repositories are available worldwide, such as the Dana-Farber Cancer Institute (DFCI) Tomato Gene Index, The SOL Genomics Network (SGN) [5], MiBASE [10], PlantGDB [11], and TomatEST [12], and the number of tomato ESTs accumulated in the databases is 296,957 (January 8, 2010). To elucidate the functions of individual genes, attempts have been made to organize ESTs into consensus sequences, which are referred to as unigenes or tentative consensus (TC) sequences. However, a more direct approach to collecting DNA information of individual genes is to analyze full-length cDNA sequences. Major advantages of sequencing full-length cDNA over the EST assembly include that the data is derived from a single clone rather than the assembly of multiple ESTs which can generate contigs containing sequences from multiple transcripts. Another major advantage of this approach is that full-length cDNA clones have complete sequences of transcripts including coding regions (CDSs) and untranslated regions (UTRs). This facilitates the subsequent annotation and prediction of genomic structures. The complete CDSs in full-length cDNAs allow the prediction of protein sequences, conserved domains, and conserved motifs. Furthermore, full-length cDNAs are easy to use with gene transfer systems, permitting the functional analysis of individual genes through reverse-genetics approaches. Thus, full-length cDNAs are a powerful genomic research tool.

In tomato genomics, a miniature tomato cultivar of *S. lycopersicum*, Micro-Tom [13], is regarded as an excellent model system. Micro-Tom has characteristics that make it suitable for experimental study, such as small size, short generation time, and ease of transformation [14,15]. Recently, resources such as various mutagenized lines have been developed in the Micro-Tom-background [16]. Thus, a collection of Micro-Tom full-length cDNAs would boost the molecular bioengineering of tomato by synergistically combining with other resources. However, no full-length cDNA collection of Micro-Tom, or of tomato, has yet been established.

In this study, we developed a large-scale collection of Micro-Tom full-length cDNAs. We constructed full-length-enriched cDNA libraries from Micro-Tom leaf, fruit, and root tissues, and we obtained full-length sequences of 13,227 cDNAs. We then checked for redundancy between sequences, for the presence of CDSs, and for the contamination of chimeric sequences, thus generating a set of 11,502 non-redundant full-length cDNA sequences (nrFLcDNAs) for sequence analysis. We report the results of UTR analyses, including length distribution, base composition; and the results of CDS analyses, including classification of functions of deduced proteins, metabolic pathway annotations, and comparisons with genes of other plants. We also report the results of mapping nrFLcDNAs onto the draft of tomato whole-genome sequence. Mapping onto genome sequence suggested that introns of tomato genes are relatively long compared to Arabidopsis and rice. We also found that the frequency of nucleotide mismatch between exon regions of Micro-Tom and those of the genome-sequencing cultivar, Heinz 1706, is 0.061%. Finally, the value of Micro-Tom full-length cDNA information will be discussed in terms of integration with the tomato genome sequence and other tomato resources.

## Results and discussion

### Construction of full-length-enriched cDNA libraries

We constructed four full-length-enriched cDNA libraries from Micro-Tom tissues including leaves, fruits, and roots. RNA samples were prepared from plants grown under 63 different conditions to increase the variation of transcripts in the libraries (see Additional file 1: Micro-Tom tissues used for RNA preparation). Leaves were treated with 9 pathogens, 5 chemical and biochemical elicitors, salicylic acid, and methyl jasmonic acid (Table 1). Leaf RNA samples were prepared from 43 tissue types under different treatment conditions (see Additional file 1: Micro-Tom tissues used for RNA preparation). Fruit RNA samples were prepared from the pericarp at four ripening stages (mature green, breaker, turning, and red ripe) harvested in the year 2003 and 2004 (Table 1, see Additional file 1: Micro-Tom tissues used for RNA preparation). Root RNA samples were prepared from plants both with and without flowers, expecting that tissues of different growth phases have different transcripts. We also prepared RNA samples from roots treated with *Fusarium oxysporum *race 2 (Table 1, see Additional file 1: Micro-Tom tissues used for RNA preparation). RNA samples prepared from leaf, fruit tissues harvested in the year 2003, fruit tissues harvested in the year 2004, and root-tissues were mixed, and the respective mixtures were used as templates for four separate cDNA libraries, designated LEFL1, FC, LEFL2, and LEFL3. The FC library was constructed by the vector-capping method [17], and the LEFL 1, LEFL2, and LEFL3 libraries were constructed by the CAP-trapper method [18]. cDNAs derived from pathogens were excluded by hybridization to mRNAs prepared from the pathogens. The subtraction process was effective, since 5'-end sequences of only 37 clones out of 109,459 randomly selected clones matched pathogen sequences in the NCBI GenBank, and these were excluded from further data processing.

**Table 1:** Micro-Tom tissues used for RNA preparation

Organ	Treatment	Tissue	Condition	Library
Leaf	CMV(strain TN) and satellite RNA	Inoculated leaves, systemic leaves	16 dpi (21-day old)	LEFL1
		
	ToMV (strain Lta1, L_11_A, L2a, Ltb1, LJB)	Inoculated leaves, systemic leaves	7, 14 dpi (28, 35-day old)	
		
	Non-pathogenic *Alternaria alternata*	Inoculated leaves	6, 24 hpi (5-week old)	
		
	*Alternaria alternate *f. sp. *lycopersici *(As-27)	Inoculated leaves	6, 24 hpi (5-week old)	
		
	*Corynespora cassiicola *(isolated from tomato and cucumber)	Inoculated leaves	6, 24 hpi (3-week old)	
		
	*Cladosporium fulvum *(strain 210, 211, 217)	Inoculated leaves	5 dpi (40-day old)	
		
	*Fusarium oxysporum *f. sp. *lycopersici *race 1 and 2	Leaves	Inoculated to root, 7 dpi (4-week old)	
		
	Non-pathogenic *Fusarium oxysporum*	Leaves	Inoculated to root, 7 dpi (4-week old)	
		
	*Pseudomonas syringae *pv. *tomato *(DC3000 wild type), pv *tabaci *isolate 6605 (wild type, *ΔfliC*, *ΔfliD*)	Spray-inoculated leaves	24 hpi (6-week old)	
		
	Flagellin from *P. syringae *pv. *tabaci *isolate 6605, fgl22	Sprayed leaves	3 hps (6-week old)	
		
	Probenazole (100 μg/ml)	Sprayed leaves	4 dps (4-week old)	
		
	Validamycin A (100 μg/ml)	Sprayed leaves	4 dps (4-week old)	
		
	Acibenzolar-S-methyl (100 μg/ml)	Sprayed leaves	4 dps (4-week old)	
		
	Validoxylamine A (100 μg/ml)	Sprayed leaves	4 dps (4-week old)	
		
	Salicilic acid (0.5 mM)	Sprayed leaves	2 dps (4-week old)	
		
	Methyl jasmonic acid (50 μM)	Sprayed leaves	2 dps (4-week old)	

Fruit	Mature green	Pericarp	40 daa, year 2003, 2004	FC/LEFL2
		
	Breaker	Pericarp	42 daa, year 2003, 2004	
		
	Turning	Pericarp	48 daa, year 2003, 2004	
		
	Red ripe	Pericarp	50 daa, year 2003, 2004	

Root	Roots from plants without flower	Roots	6, 10-week old	LEFL3
		
	Roots from flowered plants	Roots	10, 12- week old	
		
	*Fusarium oxysporum *race 2	Roots	1, 7, 14, 21 dpi, 4- to 11-week old	

Abbreviations: dpi, days post inoculation; hpi, hours post inoculation; hps, hour post spray; dps, days post spray; daa, days after anthesis. More detailed dexcription of the samples is available as Additional file 1.

### Full-length sequencing of cDNAs

**Figure 1:**
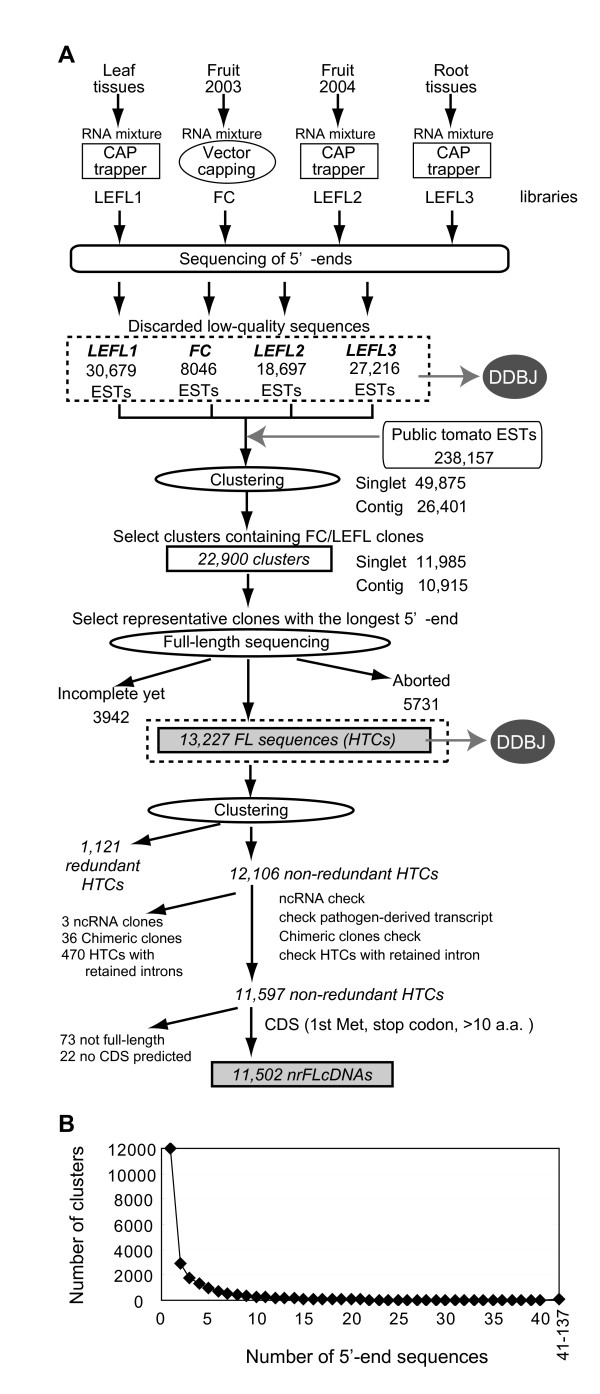
**Data processing scheme of tomato full-length cDNA sequences**. (A) Scheme for data processing of tomato full-length cDNA sequences. Four separate full-length-enriched libraries, LEFL1, FC, LEFL2, and LEFL3, were constructed. From randomly chosen clones, we obtained high-quality 5'-end sequences, 30,679, 8046, 18,697, and 27,216 sequences from the LEFL1, FC, LEFL2, and LEFL3 libraries, resprectively. These high-quality 5'-end sequences were registered in the EST division of the DDBJ. These were combined with 238,157 public tomato ESTs and then clustered into 76,276 groups. Clusters containing FC or LEFL sequences as a member were selected. The FC or LEFL sequence with the longest 5'-extension was chosen as the representative of each cluster and sent for full-length sequencing. Full-length sequencing was finished for 13,227 cDNAs, which were registered in the high throughput cDNA (HTC) division of the DDBJ. From 13,227 HTCs, 12,106 non-redundant full-length cDNAs were chosen. The 12,106 full-length cDNA set was tested for whether it contained non-coding RNA-derived cDNAs, pathogen transcript-derived cDNAs, chimeric clones, and cDNAs containing retained introns. After excluding these sequences, a set of 11,597 non-redundant HTCs was checked for CDS. Finally, a set of 11,502 non-redundant full-length cDNAs (nrFLcDNAs) was generated for subsequent sequence analyses. (B) Distribution of the number of 5'-end sequences derived from FC and LEFL cDNA libraries in each cluster.

A schematic flow of the sequencing process is presented in Figure 1A. We randomly selected 109,422 independent clones from leaf, fruit, and root libraries, and sequenced them from the 5'-end. After trimming vector-derived sequences and low-quality sequences, 89,872 5'-end sequences (30,679, 8046, 18,697 and 27,216 sequences from the LEFL1-, FC-, LEFL2- and LEFL3 libraries, respectively) were combined with Micro-Tom ESTs reported previously [9] and tomato ESTs registered in the SGN database. A total of 322,813 ESTs were grouped into 76,276 clusters. From 76,276 clusters, 22,900 clusters containing FC and LEFL clones were selected. The number of FC or LEFL clones in each cluster ranged from 1 to 137 (Figure 1B). The FC or LEFL sequence having the longest 5'-end extension in each cluster was selected as the representative of that cluster.

Full-length sequencing was performed using the representative clones by the clone-by-clone primer walking method. Out of 22,900 clones, full-length inserts of 13,227 cDNA clones were sequenced (see Additional file 2: Clone number list). Mean of phred quality value of each base was 65 (i.e., one error in 3 × 10^6 ^bases), and more than 70% of bases exceeded quality value 68. This collection was registered to the high-throughput cDNA sequence (HTC) division of the DDBJ.

### Generation of a non-redundant set of the full-length cDNAs

We checked for redundancy within the set of 13,227 HTCs, and then a set of 12,106 HTCs representing non-redundant transcripts was sent for further processing (see Additional file 2: Clone number list). To check whether each of the 12,106 HTCs has a full-length CDS or not, we first searched for cDNAs derived from non-coding RNAs using the NONCODE version 2.0 dataset [19]. Three HTCs (FC25DB10, LEFL3003O13, and LEFL3054J22) were identified as cDNAs derived from non-coding RNA (see Additional file 3: cDNA derived from ncRNA) and excluded from further analysis. Next, we confirmed that all of the HTCs did not match DNA sequences from pathogens used to attack leaf and root tissues, suggesting that the probability of contamination with pathogen-derived transcripts was negligible (see Additional file 2: Clone number list, for the accession numbers of pathogen-derived sequences). We then checked for chimeric clones putatively generated during cDNA cloning process. According to the criteria described in Methods, 36 HTCs were regarded as chimeric clones (see Additional file 2: Clone number list). We then identified sets of HTCs generated by the alternative splicing event, and excluded 470 HTCs containing retained introns (see Additional file 2: Clone number list). From the remaining 11,597 HTCs, CDSs were predicted by using FrameDP [20] and GeneMark.hmm-E [21] programs, and by selecting open reading frame encoding the longest amino acid sequence. Essentially, a CDS encoding amino acid sequence that had the highest similarity to proteins registered in either the nr or the tomato SBM protein databases was selected as representative CDS of the HTC (Additional file 4: Predicted CDS of 11,597 non-redundant HTCs). HTCs with CDSs shorter than nine amino acid (aa)long were excluded from further analysis, since the shortest length tomato protein registered in UniProt was 10 aa (UniProt accession number, Q6TS30; description, ENOD40). Out of 11,597 non-redundant HTCs, CDS encoding full-length protein was predicted for 11,502 HTCs. Full-length CDS were not predicted for 73 HTCs and no coding regions were predicted for 22 HTCs. Finally, a set of 11,502 HTCs (see Additional file 2: Clone number list) was generated, hereafter referred to as nrFLcDNA. This nrFLcDNA set was subjected to subsequent sequence analyses.

**Figure 2:**
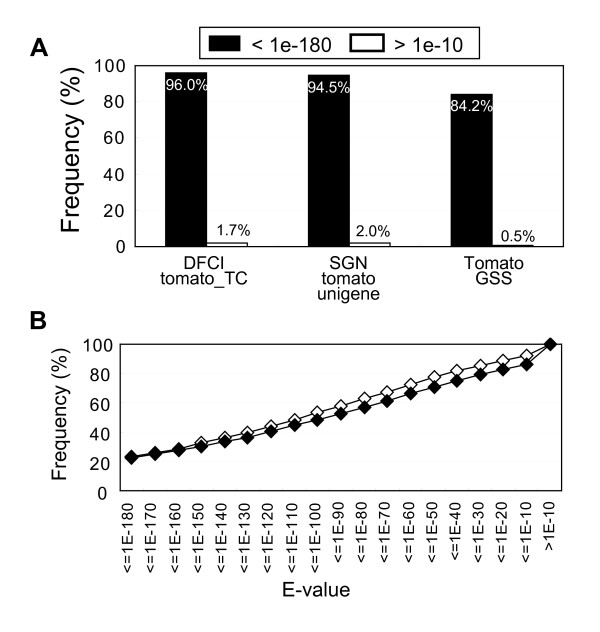
**Similarity of nrFLcDNAs with public tomato sequences**. (A) Similarity of the 11,502 nrFLcDNAs to DFCI tomato tentative consensus (TC), SGN tomato unigene, and the prerelease of tomato genome shotgun sequence (Tomato GSS). Black bars: nrFLcDNAs that showed very high similarity (E-value < 1e-180), white bars; nrFLcDNAs that showed very low similarity (E-value > 1e-10). (B) Similarity of amino acid sequences predicted from the 11,502 nrFLcDNAs to proteins in nr (white rectangle) and the Tomato SBM database (black rectangle).

Nucleotide sequences of 11,502 nrFLcDNAs were compared with DFCI Tomato Gene Index release 12.0 tentative consensus (TC), SGN tomato unigenes (SGNtomato_20090805), and the prerelease of tomato genome shotgun sequence (S_lycopersicum_scaffolds_20091201) (Figure 2A). Percentage of nrFLcDNAs that did not match (E-value greater than 1e-10) was 1.7%, 2.0%, and 0.5% against DFCI tomato TCs, SGN tomato unigenes, and tomato genome sequences, respectively. Predicted amino acid sequences of 11,502 HTCs (Additional file 4: Predicted CDS of 11,597 non-redundant HTCs) were compared with proteins registered in NCBI nr dataset and Tomato SBM protein dataset (protein_sequence, ftp://ftp.kazusa.or.jp/pub/tomato/) (Figure 2B). Distribution of E-value was similar against both datasets. Approximately 22% of the nrFLcDNAs had very high similarity (E-value < 1e-180) to nr and SBM proteins, and more than 75% of the nrFLcDNAs have E-value smaller than 1e-50.

### Length distributions of UTRs and CDSs

**Figure 3:**
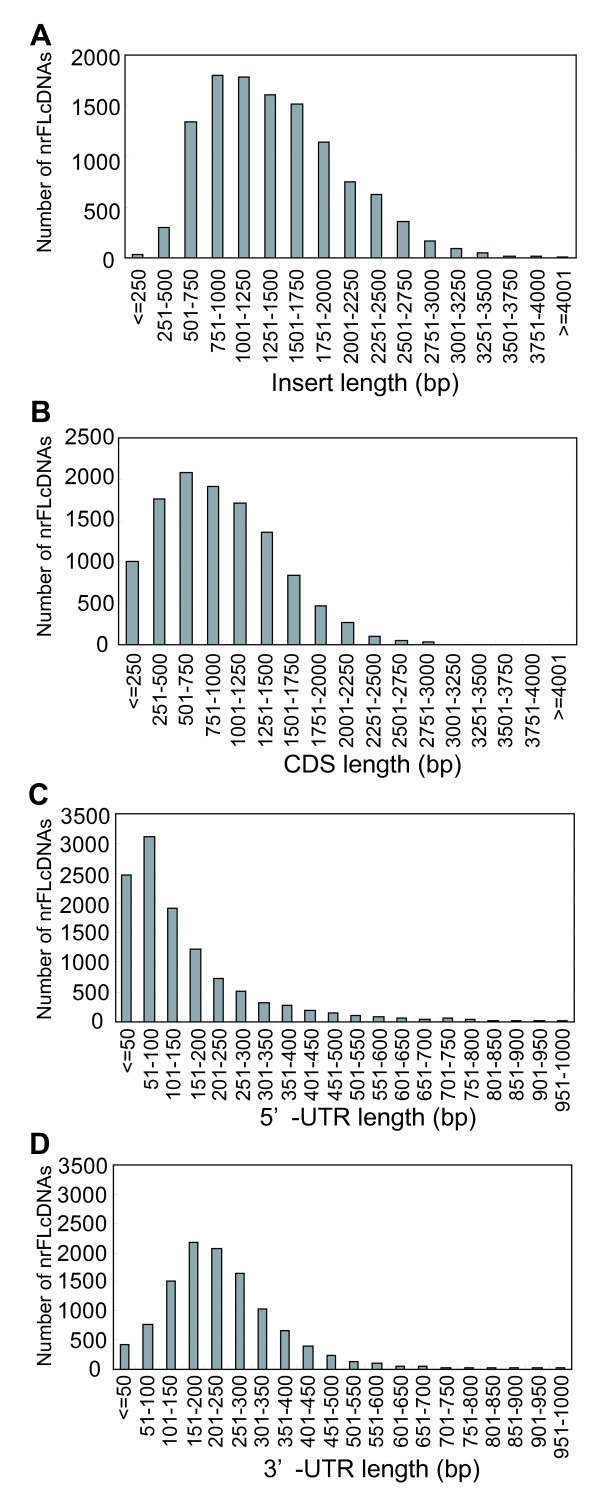
**Distribution of characteristic lengths of nrFLcDNAs**. (A) cDNA insert length, (B) CDS length, (C) 5'-UTR length, and (D) 3'-UTR length. 5'- and 3'-UTR lengths were longer than those of Arabidopsis, soybean, poplar, and maize, and slightly shorter than those of rice.

Figures 3A and 3B show the distributions of cDNA insert length and the CDS length, respectively. The average insert length was 1418 bp, which is shorter than those of Arabidopsis (1445 bp [22]) and soybean (1539 bp [23]) (Table 2). The median value for the insert length was 1324 bp, which is shorter than Arabidopsis (1459 bp) and rice (1548 bp) [24], but longer than poplar (990 bp [25]) (Table 2). The average length of the CDS was 938 bp, corresponding to an average polypeptide length of 313 aa, which was also shorter than those of Arabidopsis, rice, and soybean, and longer than poplar and maize [26,27] (Table 2). The nrFLcDNA set contains 904 sequences derived from the FC clones that were cloned by the vector-capping method, and these harbor shorter cDNA inserts (average insert length 773 bp, average CDS length 482 bp) than those derived from the LEFL clones that were cloned by the CAP-trapper method (average insert length 1474 bp, average CDS length 977 bp). This probably explains why the average lengths are shorter than those reported for full-length cDNAs of other plants produced by the CAP-trapper method.

**Table 2:** Length of cDNA inserts, CDSs, and UTRs, and comparison with full-length cDNAs from other plants

		cDNA insert	CDS	5'-UTR	3'-UTR	Reference
Tomato	average	1418	938	175	257	(Present study)
	median	1324	876	106	220	

Arabidopsis	average	1445	n.a.	n.a.	n.a.	[24]
	median	1459	1097	88	184	

Soybean	average	1539	1042	123	248	[23]
	median	n.a.	933	75	233	

Poplar	average	1045	649	109	228	[25]
	median	990	558	77	209	

Rice	average	n.a.	993	260	398	[24]
	median	1548	947	123	279	

Maize	average	n.a.	799	99	206	[26]
	median	n.a.	741	80	228	[27]

n.a.: Not available.

Figures 3C and 3D show the distributions of the 5'- and 3'-UTR lengths, respectively. In contrast to CDS length, tomato nrFLcDNAs had longer UTRs compared to those of other plants. The average 5'- and 3'-UTR lengths were 175 and 257 bp, respectively. The median 5'- and 3'-UTR lengths were 106 and 220 bp, respectively. On both average and median bases, the UTRs of tomato are longer than those of Arabidopsis, soybean, poplar, and maize, and slightly shorter than those of rice (Table 2). The relatively long UTRs of tomato likely contribute to the regulation of mRNA transcription, translation, and stability [28].

### Nucleotide composition

The nucleotide composition of nrFLcDNAs differed among the 5'-UTRs, CDSs, and 3'-UTRs (Table 3). The AT-content of the full-length cDNAs was 59.7%, which is slightly higher than that of Arabidopsis full-length cDNAs (57%) [24]. AT richness was more pronounced in the 5'- and 3'-UTRs (63.5% and 66.3%, respectively), and less pronounced in the CDSs (57.2%). The 5'-UTRs had more Cs than the 3'-UTRs, and the number of Ts was higher in the 3'-UTRs than in the 5'-UTRs. These results are similar to those observed in Arabidopsis full-length cDNAs [24]. However, nucleotide compositions of the nrFLcDNA was more AT-rich than those of maize transcripts [27]. The most frequently used stop codon is TGA (occur 40.6% of all nrFLcDNAs) followed by TAA (35.6%) and TAG (23.8%). Frequency of stop codon is similar to that occurred in Arabidopsis (TGA, 44%; TAA, 36%; and TAG, 20%), but different from those occurred in rice (TGA, 43%; TAA, 27%; and TAG, 30%) and maize (TGA, 51%; TAA, 19%; and TAG, 30%) [27].

**Table 3:** Nucleotide composition for different parts of tomato full-length cDNAs

	Frequency (%)			
	
	A	T	G	C
				
Full-length cDNA	29.3	30.4	21.7	18.6
5'-UTR	30.2	33.3	17.1	19.4
CDS	29.1	28.1	23.4	19.4
3'-UTR	29.3	37.0	18.3	15.4
				

### Alternative splicing and retained intron

**Figure 4:**
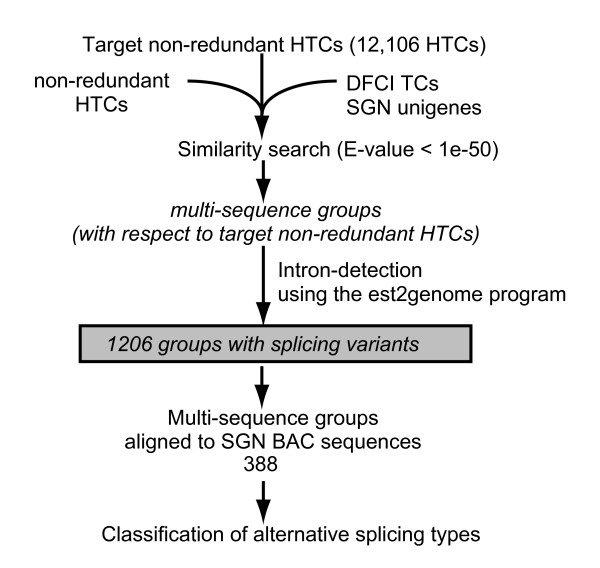
**Scheme for detection of alternatively spliced transcripts**. Each nrFLcDNA was searched against nrFLcDNA dataset, DFCI tomato TCs, and SGN tomato unigenes using BLASTN. Query nrFLcDNAs were designated as "target nrFLcDNAs." Sequences that matched the target nrFLcDNA by a threshold E-value of 1e-50 were grouped into a multi-sequence group. Intron detection was performed using the est2genome program by setting the target nrFLcDNA as "est" and member nrFLcDNA, unigene, or TC as "genome", and vice versa. Consequently, splicing variants were detected for 1,206 target nrFLcDNAs. Out of the 1,206 nrFLcDNAs, 388 nrFLcDNAs were aligned to SGN tomato BAC sequences (bacs.v374.seq.20081128091837). Classification of alternative splicing events was carried out manually using the 388 multi-sequence groups.

As reported for Arabidopsis and rice full-length cDNA collections, the collection of full-length cDNAs contains cDNAs generated from alternatively spliced transcripts [29,30]. To identify nrFLcDNAs derived from alternatively spliced transcripts, we first generated multi-sequence groups using the non-redundant 12,106 HTCs, SGN tomato unigenes, and DFCI tomato TCs according to the similarity to the target non-redundant HTCs (Figure 4). All pairs of target non-redundant HTCs and member sequences were subjected to intron detection, and we confirmed that 1206 target non-redundant HTCs had splicing variants. The percentage of non-redundant HTCs with splicing variants was 10%, which is slightly lower than that of Arabidopsis (11.6%) [29] or rice (13.1%) [30].

Out of these 1206 non-redundant HTCs, 388 HTCs were aligned with SGN tomato BAC sequences (bacs.v374.seq.20081128091837 downloaded from the SGN ftp site http://sgn.cornell.edu/bulk/input.pl?mode=ftp), which allowed us to classify the types of 434 alternative splicing events (see Additional file 2: Clone number list). The retained intron-type was the most frequent (55% of all events). The alternative acceptor site- and alternative donor site-types also occurred frequently, comprising 20% and 16% of all events, respectively. The exon skip-type and other types (e.g., alternative terminal exon) were rather rare. The occurrence of alternative acceptor site- and alternative donor site-types was slightly higher than that reported previously for tomato [31]. nrFLcDNAs with retained introns encode proteins with alternative amino acid sequences. We found that 509 nrFLcDNAs may contain retained full- or partial-length introns (see Additional file 2: Clone number list). Larger scale identification of alternative splicing events using the draft of tomato whole-genome sequence is currently underway.

### Functional annotation of tomato full-length cDNA

**Figure 5:**
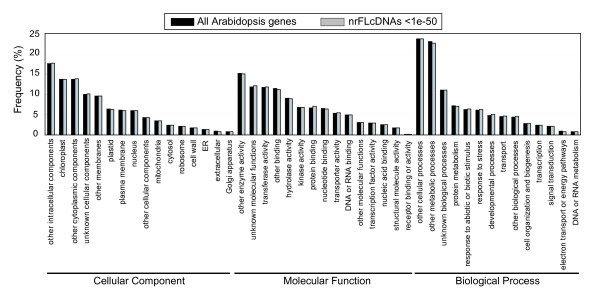
**Profile of GO annotations for nrFLcDNAs**. To obtain GO annotations for nrFLcDNAs, similarity between the amino acid sequences predicted from nrFLcDNAs and Arabidopsis proteins was assessed using BLASTP. Arabidopsis genes corresponding to the top hit Arabidopsis protein to each nrFLcDNA was chosen at threshold E-values of 1e-50 (gray bars). GO annotations for nrFLcDNAs were then retrieved by subjecting the list of Arabidopsis genes to a TAIR GO annotation search http://www.arabidopsis.org/tools/bulk/go/index.jsp. GO annotations for all Arabidopsis genes (black bars) were retrieved from the TAIR GO annotation search. No statistically significant difference in the frequency was observed in all categories.

Next, putative functions of nrFLcDNAs were assessed by BLASTP searches against the TAIR9, RAP-DB, and NCBI nr protein datasets. To identify functions of the nrFLcDNAs, we used gene ontology (GO) annotations for Arabidopsis genes that matched nrFLcDNAs. The results from the case using Arabidopsis proteins that match nrFLcDNAs at E-value smaller than 1e-50 is shown (see Additional file 2: Clone number list). This revealed that nrFLcDNAs covered functional categories as broadly as all Arabidopsis genes (Figure 5).

**Figure 6:**
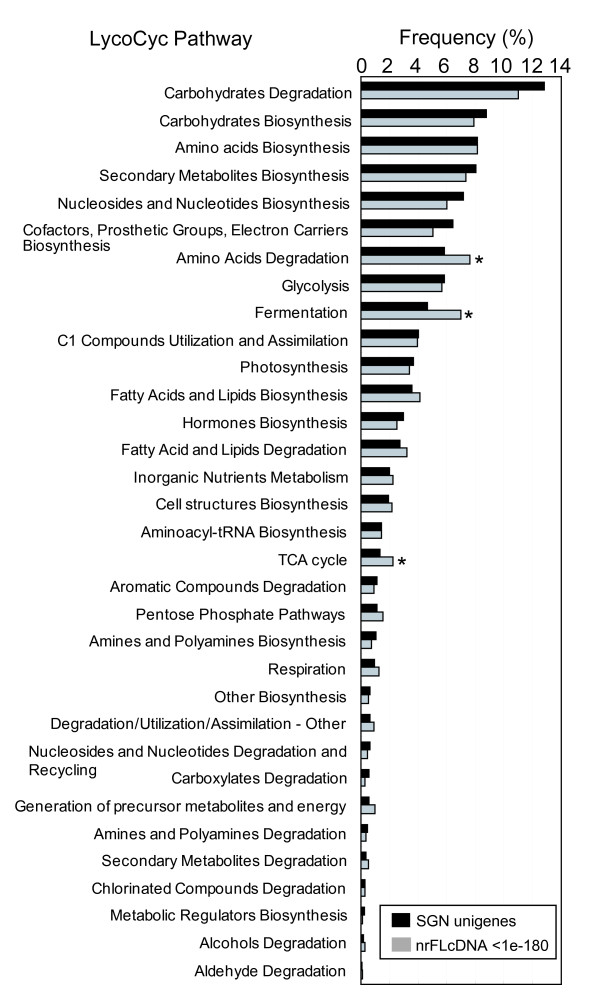
**Profile of metabolic pathway annotations for the nrFLcDNAs according to LycoCyc**. To obtain LycoCyc annotations for the nrFLcDNAs, similarity between nrFLcDNAs and SGN tomato unigenes was assessed using BLASTN. The top hit SGN tomato unigene to each nrFLcDNA was extracted at a threshold E-value of 1e-180 (gray bars). LycoCyc pathway annotations for the top hit unigenes were then regarded as annotations for nrFLcDNAs. Pathway annotations for all SGN tomato unigenes (black bars) was retrieved from documents of LycoCyc ver 1.0.1.1. Asterisks indicate that the difference in the frequency is statistically significant at 5% level.

To obtain insights into the metabolism-related genes, the nrFLcDNAs were assigned to the LycoCyc pathways http://sgn.cornell.edu/tools/solcyc/. Based on the best matches to SGN tomato unigenes, 448 nrFLcDNAs (see Additional file 2: Clone number list) were assigned to LycoCyc pathways, and 1117 pathway annotations were obtained. Comparison of the pathway-classification patterns with those for all SGN unigenes demonstrated that the relative abundance of annotations assigned to the pathways "Amino Acids Degradation", "Fermentation", and "TCA cycle" was higher in the nrFLcDNAs than in all SGN unigenes (Figure 6). This demonstrated that genes related to primary metabolism are slightly overrepresented in the nrFLcDNA set compared to genes related to secondary metabolism.

We then attempted to identify transcription factors that are crucial for the transcriptional regulation of various biological processes. We searched for transcription factor domains described in AGRIS AtTFDB [32] using the InterPro database by querying the CDS of the nrFLcDNAs. This search demonstrated that 311 nrFLcDNAs (see Additional file 2: Clone number list) had 417 InterPro entries classified into 34 transcription factor families (Table 4). The G2-like and MYB families were the most numerous (the nrFLcDNAs assigned to the MYB family were completely overlapped with those in the G2-like family), followed by the Homeobox family. This result was slightly different from those of Arabidopsis [33] and rice [30], in which the AP2/EREBP family and zinc finger family were predominant, respectively. Difference in the distribution of transcription factors between tomato nrFLcDNA and Arabidopsis implies that the nrFLcDNA set failed to contain transcription factors whose expression level is low or cell type-specific.

**Table 4:** Transcription factors found in the nrFLcDNAs

Family	IPR entry	Description of IPR entry	No. of tomato nrFLcDNAs
G2-like	IPR006447	Myb-like, SHAQKYF class	52^a^
MYB	IPR015495	Myb transcription factor	50^a^
Homeobox	IPR001356	Homeobox	40
C2H2	IPR007087	Zinc finger, C2H2-type	31
bHLH	IPR011598	Helix-loop-helix DNA-binding	22
CCAAT-HAP5, HAP3, and HAP2	IPR003958	Transcription factor CBF/NF-Y/archaeal histone	19
PHD	IPR001965	Zinc finger, PHD-type	18
AP2-EREBP	IPR001471	Pathogenesis-related transcriptional factor and ERF, DNA-binding	18
WRKY	IPR003657	DNA-binding WRKY	18
bZIP	IPR011616	bZIP transcription factor, bZIP-1	17
HSF	IPR000232	Heat shock factor (HSF)-type, DNA-binding	15
C3H	IPR000571	Zinc finger, CCCH-type	13
MADS	IPR002100	Transcription factor, MADS-box	11
MYB-related	IPR010588	Myb-related protein P, C-terminal	11
C2C2-YABBY	IPR006780	YABBY protein	11^b^
ARID	IPR009071	High mobility group box	11^b^
NAC	IPR003441	No apical meristem (NAM) protein	10
TCP	IPR005333	Transcription factor, TCP	8
GRAS	IPR005202	GRAS transcription factor	7
C2C2-GATA	IPR000679	Zinc finger, GATA-type	6
ABI3VP1	IPR003340	Transcriptional factor B3	6
EIL	IPR006957	Ethylene insensitive 3	3
BBR/BPC	IPR010409	GAGA binding-like	2
TUB	IPR000007	Tubby, C-terminal	2
C2C2-Dof	IPR003851	Zinc finger, Dof-type	2
C2C2-CO-like	IPR000315	Zinc finger, B-box, "IPR002926 Zinc finger, CONSTANS-type" was deleted from IPR entry.	2
ZF-HD	IPR006456	ZF-HD homeobox protein Cys/His-rich dimerisation region	2
GeBP	IPR007592	Protein of unknown function DUF573	2
ARR-B	IPR001789	Response regulator receiver	2
NLP	IPR003035	Plant regulator RWP-RK	2
BZR	IPR008540	BZR1, transcriptional repressor	1
JUMONJI	IPR013129	Transcription factor jumonji	1
ZIM	IPR007853	Zinc finger, Zim17-type	1
Whirly	IPR009044	ssDNA-binding transcriptional regulator	1

^a^Member nrFLcDNAs of MYB were completely overlapped with those of G2-like. ^b^Member nrFLcDNAs of C2C2-YABBY and ARID completely overlapped.

### Comparative analysis with genes of other plants

**Figure 7:**
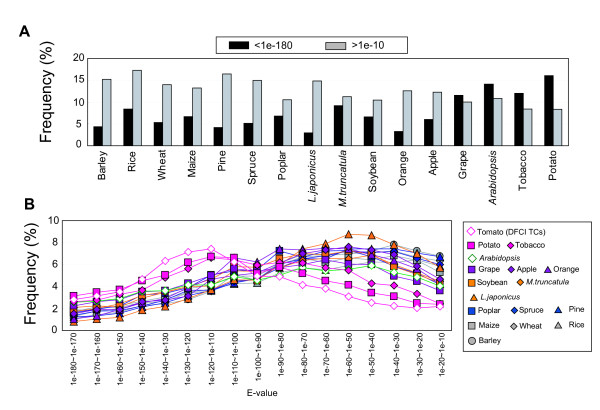
**Comparison of nrFLcDNAs with genes of other plants**. (A) Percentage of nrFLcDNAs with very high similarity (E-value < 1e-180, black) and very low similarity (E-value > 1e-10, gray) to genes of other plants. (B) Distribution of the number of nrFLcDNAs matched plant unigenes with intermediate similarity between 1e-180 and 1e-10.

To assess the similarity of nrFLcDNAs with genes of other plants, deduced peptide sequences were compared with protein databases of Arabidopsis (TAIR9) and rice (RAP-DB) using BLASTP, and with the DFCI Gene Indices of barley, wheat, maize, pine, spruce, poplar, *Lotus japonicus*, *Medicago truncatula*, soybean, orange, apple, grape, tobacco, and potato using tBLASTN (see Additional file 5: Datasets used for comparison with other plants). nrFLcDNAs has high similarity to two solanaceae plants, potato and tobacco (Figure 7A). Percentages of nrFLcDNA that did not match to potato and tobacco TCs (E-value > 1e-10) were 8.4% and 8.5%, respectively, which are the lowest among these 16 species. On the other hand, percentages of nrFLcDNA that did not match (E-value > 1e-10) to barley, rice, wheat, maize, pine, spruce, and *L.japonicus *were higher than the average over these 16 species (12.6%). Distribution patterns of nrFLcDNAs in varied E-value range between 1e-180 and 1e-10 were classified into two groups (Figure 7B). Distribution curves of nr FLcDNAs matched potato and tobacco TCs have peaks at 1e-100 and are similar to the distribution of nrFlcDNAs matched DFCI tomato TCs. On the other hand, distribution curves of nrFLcDNAs matched TCs of other 14 plants have peaks at 1e-50 or larger. These results suggest that tomato transcript sequences are more closely related to transcripts from potato and tobacco than to other plant species.

To obtain insight into functions of nrFLcDNAs that were not found in other plants, we extracted 694 nrFLcDNAs that matched none of the other plant genes (see Additional file 2: Clone number list). More than 90% of the 694 nrFLcDNAs matched the prerelease of tomato genome shotgun sequence with E-value smaller than 1e-120 and only 27 nrFLcDNAs did not match genome sequence with E-value threshold of 1e-10, suggesting that most of the 694 nrFLcDNAs are tomato-derived transcripts. By searching the InterPro database, out of the 694 nrFLcDNAs, 54 nrFLcDNAs were assigned with 75 protein domains. We also identified known protein domains by searching the similarity with the tomato SBM protein datasets. Out of 48 nrFLcDNAs matched (E-value < 1e-10) SBM proteins, 19 nrFLcDNA had known protein domains. Domains such as Cyclin-like F-box (IPR001810), Aldehyde dehydrogenase (IPR015590), and Polynucleotidyl transferase, Ribonuclease H fold (IPR012337) were identified in both searches. However, known functional protein domains were not found in most (640 out of 694) of the nrFLcDNAs that were not found in other 16 plants.

### Mapping full-length cDNAs onto tomato genomic sequence

**Figure 8:**
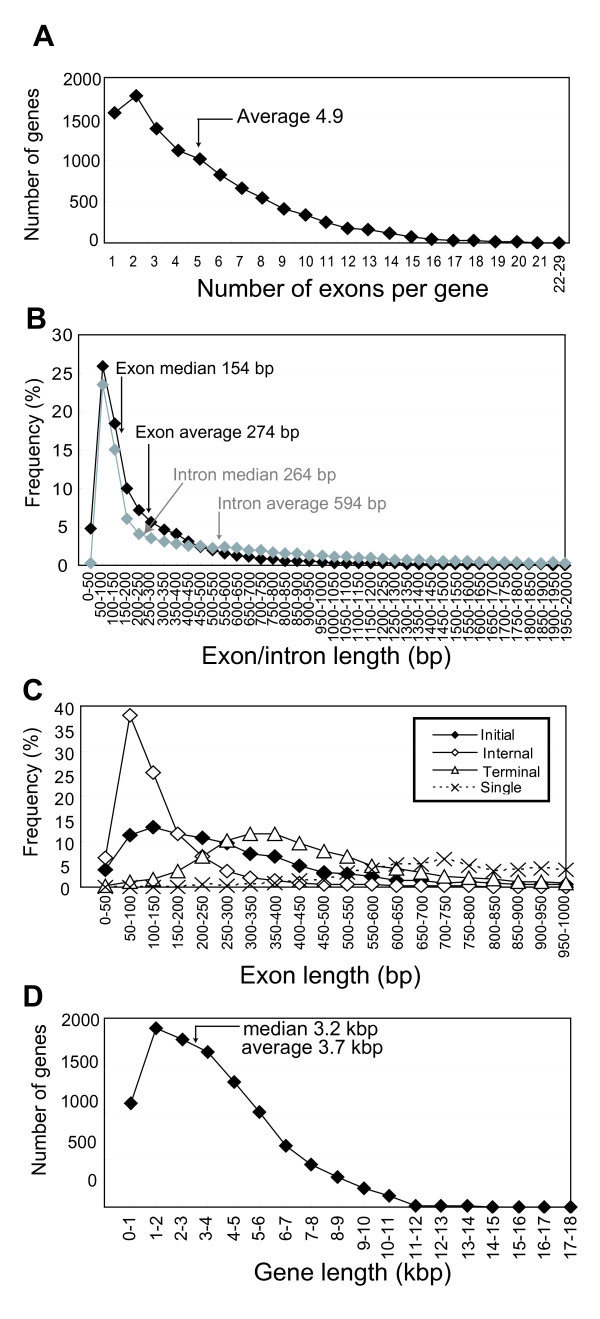
**Distribution of the number and lengths of exons and introns**. (A) Number of exons per gene. (B) Distribution of exon length (black curve) and intron length (gray curve). (C) Distribution of lengths of the initial exons (black rectangle), the internal exons (white rectangle), the terminal exons (white triangle), and the single exons (dotted line). (D) Distribution of gene length.

Mapping of full-length cDNA sequences onto the genome sequence provides insights into tomato genomic structure. Out of 11,502 nrFLcDNAs, full-length regions of 10,544 nrFLcDNAs (see Additional file 2: Clone number list) were mapped onto the prerelease of tomato genome shotgun sequence (S_lycopersicum_scaffolds_20091201). The rest, 958 nrFLcDNAs, failed to match genome sequence with nucleotide identity larger than 90% or with alignment length more than 90% of the nrFLcDNA, or failed to match in full-length. Based on this full-length mapping, the average exon number per gene was estimated to be 4.9 (median value, 4 exons per gene) (Figure 8A). Exon length ranged from 14 to 4528 bp. The average and median exon lengths were estimated to be 274 bp and 154 bp, respectively (Figure 8B, black curve). Intron lengths showed larger variation than exon lengths, ranging from 12 to 9664 bp. The average and median intron lengths were estimated to be 594 bp and 264 bp, respectively (Figure 8B, gray curve). The median intron length was longer than those of Arabidopsis (100 bp) and rice (145 bp). To compare exon lengths with those of Arabidopsis and rice, we classified exons into four types: initial, internal, terminal, and single exons. The distribution profiles of exon lengths were similar to those observed in Arabidopsis (Figure 8C) [24]. The median lengths of the initial, internal, and terminal exons were 244, 109, and 415 bp, respectively, which were comparable to those of Arabidopsis (274, 112, and 402 bp) and rice (284, 113, and 476 bp). Finally, we estimated the entire length of each gene corresponding to nrFLcDNA. The average and median lengths of tomato genes were estimated to be 3735 bp and 3281 bp, respectively (Figure 8D).

To investigate the occurrence of introns in UTRs, the positions of translation start sites and translation termination sites were mapped to the exons. The frequency of the first methionine in the internal exons was 19.9%. On the other hand, the frequency of stop codons in the internal exons was 5.7%. This result indicates that intron mapping to a UTR was more likely to occur in 5'-UTRs than in 3'-UTRs.

This mapping also provides insights into the single nucleotide polymorphism frequency between Micro-Tom and the genome-sequencing cultivar, Heinz 1706. To estimate frequency of nucleotide mismatch, we used nrFlcDNA-scaffold pairs with identity in the BLAST output equal to or larger than 99.5%. Resulting set of nrFlcDNA-scaffold pairs contained 90% of the total pairs. Frequency of nucleotide mismatches in exon regions was estimated to be 0.061% (i.e., one difference in 1640 nt). This is nearly comparable to the previous result based on the comparison of ESTs between Micro-Tom and other *S. lycopersicum *cultivars [9]. This result suggests that the Micro-Tom cDNA sequence serves as a good reference for the tomato genome sequence. We note that the results presented here will be revised when annotation of the tomato genome sequence is finished in near future.

### A tomato full-length cDNA database: KaFTom

Information regarding the13,227 full-length cDNA sequences including annotations and the results of similarity searches are distributed from the KaFTom database http://www.pgb.kazusa.or.jp/kaftom/. KaFTom also provides the results of mapping full-length sequences onto SGN tomato BAC sequences and predictions of exons and introns. It directly links to the clone request site, and all of the full-length cDNA clones (89,872 clones) are available from the National Bioresource Project Tomato http://tomato.nbrp.jp.

### Significance of Micro-Tom full-length cDNA in tomato genomics

Full-length cDNAs serves as a valuable tool that will accelerate tomato genomics in several respects. First, the sequence information of the full-length cDNAs will be integrated into the whole-genome sequence information of tomato, and it will help genome annotation and the identification of regulatory elements in UTRs. Second, sequences of the full-length cDNAs are valuable information for generating molecular markers. Third, full-length cDNAs help our understanding of the functions of tomato genes through gain-of-function and loss-of-function analyses. Fourth, full-length cDNAs promote the use of "targeting induced local lesions in genome" (TILLING) screening. By combining the use of full-length cDNA sequence information with EMS-mutagenized lines of Micro-Tom [16], the TILLING approach will provide an efficient way to screen non-transgenic mutant lines as parental germplasm favorable for breeding programs.

## Conclusion

We developed a set of 13,227 full-length cDNAs from the model tomato cultivar Micro-Tom, and then we generated a set of 11,502 nrFLcDNAs, each of which represents a non-redundant transcript coding a full-length CDS. Analysis of the 5'-UTRs, CDSs, and 3'-UTRs demonstrated that tomato transcripts have longer 5'- and 3'-UTRs than other plant species. Classification of functions of deduced proteins according to the GO annotation revealed that the nrFLcDNA set covers a broad range of proteins. Comparison of the nrFLcDNAs with genes of other plants facilitated the identification of tomato cDNAs that have very low similarity to 16 other plants tested, most of which did not have known protein domains. Mapping of the nrFLcDNAs onto tomato genome sequences revealed that tomato genes have longer introns than Arabidopsis and rice. The frequency of single nucleotide polymorphisms between exons of Micro-Tom and the genome-sequencing cultivar, Heinz 1706, was estimated to be 0.061%. In combination with other resources, the Micro-Tom full-length cDNAs will bridge the gap between basic and applied studies, providing a valuable tool not only for tomato whole-genome annotation but also for genomics studies and molecular breeding.

## Methods

### Plant materials

The miniature tomato cultivar, Micro-Tom (*Solanum lycopersicum *cv Micro-Tom), was used to construct full-length cDNA libraries. Total RNA samples were prepared from leaves, fruits, and roots of Micro-Tom. The tissues used for RNA preparation are summarized in Table 1. For a more detailed description of tissue samples, see Additional file 1: Micro-Tom tissues used for RNA preparation.

Leaves were treated with various pathogens. For fungal and bacterial inoculation, Micro-Tom plants were grown in sterilized soil at 20-28°C with a photoperiod of 14-16 h light (7000 lux, or 85 μmol/m^2 ^s)/8-10 h dark. For virus inoculation, Micro-Tom plants were grown in a mixture of vermiculite and perlite (1:1 (v/v)) at 23-25°C with a photoperiod of 16 h light (8000 lux, or 97 μmol/m^2 ^s)/8 h dark, and irrigated with a nutrient medium every three days. Pathogen treatments were performed as described previously [34].

Fruits were harvested at four ripening stages, mature green, breaker, turning, and red ripe stages. We used two harvests of Micro-Tom fruit for fruit RNA preparation. In the first harvest, Micro-Tom plants were grown in the year 2003 and fruit pericarp was harvested as described previously [35]. In the second harvest, Micro-Tom plants were grown in the year 2004 and fruit pericarp was harvested as described previously [36].

Roots were harvested from plants before and after flowering, and from plants treated with *Fusarium oxyporum *race 2. For root samples from plants before and after flowering, Micro-Tom seeds were sterilized with 70% (v/v) ethanol for 1 min, and germinated on sterile MS-agar plates (3% (w/v) sucrose) in a photoperiod of 12 h light (7000 lux, or 85 μmol/m^2 ^s)/12 h dark at 26°C. On the 10^th ^day after germination, seedlings were transferred to a sterile mixture of vermiculite and Powersoil (1:2) (Kureha Chemical Industries, http://www.kureha.co.jp/ and Kanto Hiryo Industries, http://www.okumurashoji.co.jp/) and were grown in a photoperiod of 12 h light (7000 lux, or 85 μmol/m^2 ^s)/12 h dark at 26°C. Roots were harvested from approximately three-month-old plants before or after flowering. For *F. oxyporum *race 2 treatment, Micro-Tom seeds were germinated on sterile soil, grown in sterile conditions, and treated with *F. oxyporum *race 2 as described previously [34].

### RNA extraction

Total RNA was extracted from the tissues using an acid guanidinium thiocyanate-phenol-chloroform method [37]. Sugars were further removed by a sodium acetate-precipitation method [35].

### Construction of the full-length-enriched cDNA library

Equal amounts of total RNA (approximately 60 μg each) from 42 leaf tissue types were mixed and then used to construct the LEFL1 cDNA library. Equal amounts of total RNA (approximately 2400 μg each) from four fruit tissues harvested in the year 2004 were mixed and then used to construct the LEFL2 cDNA library. Likewise, equal amounts of total RNA (approximately 230 μg each) from 12 root tissue types were mixed and then used to construct the LEFL3 cDNA library. Construction of the above-mentioned libraries was performed by the biotinylated CAP-trapper method as described previously [18]. For the RNA mixture from fruits harvested in the year 2003, a full-length-enriched cDNA library, namely the FC library, was constructed by the vector-capping method described previously [35]. Finally, cDNA inserts were cloned into the pFLCIII vector or the pGCAPzf3 vector.

### 5'-end sequencing and clustering

cDNA clones randomly collected from the libraries were single-pass sequenced from the 5'-end as described previously [35]. From the 5'-end sequences, vector-derived sequences and low-quality regions (Phred quality value < 30) were trimmed using a combination of the cross_match program http://www.phrap.org and the phred program [38]. Sequence data of lengths shorter than 50 bases were not included in further data processing. If the repetition of a single nucleotide was longer than 10% of a sequence, such sequences were not included in further data processing. The 5'-end sequences were combined with previously sequenced Micro-Tom ESTs [9] and ESTs registered in the SGN database (file name: tomato_species_2008_10_21.seq, obtained from the SGN ftp site ftp://ftp.sgn.cornell.edu/est_sequences/species/Tomato/), then clustered using the phrap program http://www.phrap.org/phredphrapconsed.html[39]. Clusters containing 5'-end sequences of clones derived from the FC and LEFL libraries were chosen. Based on the sequence alignment within each cluster, an FC or LEFL clone carrying the insert that has the longest extension in the 5'-direction was selected as a representative of the cluster. The representative clones were sent for full-length sequencing. All of the 5'-end sequences were registered to the DDBJ http://www.ddbj.nig.ac.jp/index-e.html with accession numbers BW684914-BW692959, DB678259-DB727670, and FS179211-206426.

### Full-length sequencing

The clones selected as the representatives of the clusters were re-arrayed in 96-well plates. A small aliquot of each clone was inoculated to 2 ml of LB medium containing 50 μg/ml ampicillin, and grown overnight at 37°C in 96-deep-well plates. Plasmid DNA was prepared from the overnight culture using CosMCPrep (Agencourt Bioscience, Beverly, MS, USA) according to the manufacturer's protocol. By using the plasmid DNA as templates, full-length sequencing was performed by a clone-by-clone primer walking method using a Model 3730 × l sequence analyzer (Applied BioSystems, Forster City, CA, USA). Sequencing reaction was performed using the BigDye Terminator version 3.1 cycle sequencing kit (Applied BioSystems). Sequences were assembled using the phrap program http://www.phrap.org/phredphrapconsed.html. Based on the assembled sequences, primers corresponding to the terminal sequences were designed using the Primer3 program [40]. The primer walking procedure was repeated until a poly(A) tail or a vector-derived sequence appeared. The full-length sequences were finished by trimming the vector-derived sequences from both ends using the cross_match program. Full-length cDNA sequences (13,227 sequences) obtained by this procedure were registered to the high-throughput cDNA sequence (HTC) division of the DDBJ with accession numbers AB211519-211522, AB211526, AK224591-AK224910, AK246135-AK248077, and AK319176-330134.

### Selection of a non-redundant set of full-length cDNAs

To identify redundant HTCs, pair-wise similarity searches between members of the 13,227 HTC sequences were performed using BLASTN. Redundant HTCs were identified using the following criteria; E-value < 1e-180, nucleotide identity ≥ 97%, and the percentage of alignment length exceeded 80% of the length of each HTC. The resulting non-redundant HTCs (12,105 HTCs) were then checked for whether they contain clones derived from non-coding RNA, clones derived from pathogen transcripts, whether they contain chimeric clones, and whether they contain clones with retained introns. HTCs derived from non-coding RNAs were identified by searching for similarity of the HTCs against the "ncrna_NONCODE[v2.0].fasta" dataset obtained from the NONCODE download site http://www.noncode.org/download.htm[19]using BLASTN with a threshold E-value of 1e-180. To search HTCs derived from pathogen-derived transcripts, nucleotide sequences from *Alternalia alternate*, *Cladosporium fulvum*, *Corynespora cassiicola*, Cucumber mosaic virus, *Fusarium oxysporum*, *Pseudomonas syringae*, and Tomato mosaic virus were retrieved from NCBI nucleotide database (see Additional file 2: Clone number list for accession numbers of pathogen-derived sequences). Similarity of the HTCs to pathogen nucleotide sequences were searched using BLASTN with threshold E-value of 1e-30. When the E-value was smaller than 1e-30, the nrFLcDNA was further checked for whether or not they have similar SGN tomato unigenes or DFCI tomato TCs with smaller E-values. If so, the nrFLcDNA was regarded as cDNA derived from Micro-Tom, not from pathogens. To identify chimeric clones, the full-length cDNA sequences were searched against SGN tomato unigenes 'draft_Solanum_lycopersicum_transcript_assembly_2009_08_05.fasta' with cut-off E-value of 1e-50. HTCs that matched more than two unigenes having different functional descriptions were identified as candidate chimeric clones. Out of these candidates, if the candidate clone matched to two or more scaffolds of the prerelease of tomato genome shotgun sequence (S_lycopersicum_scaffolds_20091201.fa) downloaded from the SGN ftp site ftp://ftp.solgenomics.net/tomato_genome/wgs/assembly, the candidate was regarded as a chimeric clone. Out of the remaining candidates, if a candidate HTC had either a BamHI or XhoI site (which was used for ligation of cDNA to vectors) in between regions matched to unigenes, the candidate was regarded as a chimeric clone. To identify HTCs containing retained intron, HTC sequences were subjected to similarity searches against the HTC itself using BLASTN with threshold E-value of 1e-50. To detect introns, pair-wise alignments of each target nrFLcDNA with members of that multi-sequence group were carried out using est2genome [41]. HTCs derived from non-coding RNAs, pathogen-derived transcripts, chimeric clones, and intron-containing clones were excluded and non-redundant 11,597 HTCs were subjected to the prediction of coding sequences.

Amino acid sequences were predicted in sense orientation using three methods. First, the CDS coding the longest amino acid sequence was predicted. Second, CDSs were predicted using FrameDP http://iant.toulouse.inra.fr/FrameDP/[20]. Parameters were set as follows: framed_minimum_peptide length, 10; reference protein database, TAIR9_pep_20090619; e-value cut-off for considering ncbi-blastx hits, 1e-3; the method used for the first classification, GC3; the maximum number of models, 3; the maximum number of iteration, 3; cut-offs for considering ncbi-blastx HSPs, e-value 1e-4 and length 100. Third, CDSs were predicted using GeneMark.hmm eukaryotic version 3.3 [21]. The model used was a_thaliana.mod which was supported by the program. Amino acid sequences of all possible frames were then subjected to BLASTP search (threshold E-value was 1e-10) against NCBI nr and the protein dataset (protein_sequence) of Tomato SBM Database http://www.kazusa.or.jp/tomato/. CDSs with the smallest E-value against proteins registered in either nr or SBM proteins were selected. If CDS with the smallest E-value did not cover full-length protein, CDS with the second smallest E-value were selected. If E-values of all CDSs were the same, the longest CDSs were selected. Whether or not HTCs have full-length CDS was assessed by checking if the amino acid sequences contain both start and stop codons. Resulting set of 11,502 non-redundant HTCs encoding full-length proteins was referred to as 'nrFLcDNA.'

### UTR identification

UTRs were identified according to the selected CDS. 5'-UTRs were defined as the nucleotide sequence upstream of the start codon. 3'-UTRs were defined as the nucleotide sequence downstream of the stop codon to the poly(A) tail. Nucleotide composition analysis was performed essentially by counting A, T, C, G, N, and X using an in-house Perl program.

### Identification of retained introns and alternative splicing

After removing poly(A) tails, a set of 12,106 non-redundant HTC sequences were subjected to similarity searches against non-redundant HTCs, the SGN tomato unigenes (Tomato_200607_build_1, ftp://ftp.sgn.cornell.edu/unigene_builds), and the DFCI Tomato Gene Index (release 12.0, http://compbio.dfci.harvard.edu/tgi/) using BLASTN with threshold E-value of 1e-50. Sequences with similarity to a given target nrFLcDNA were grouped into a multi-sequence group. To detect introns, pair-wise alignments of each target non-redundant HTCs with members of that multi-sequence group were carried out using est2genome. Intron detection was performed bi-directionally, that is, nrFLcDNA/TC/unigene and a target nrFLcDNA were set as "genome" and "est," and vice versa. A BAC sequence file named "bacs.v374.seq.20081128091837" was downloaded from the SGN ftp site http://sgn.cornell.edu/bulk/input.pl?mode=ftp. The target HTCs were mapped onto the BAC sequence with the threshold E-value of 1e-50.

### Annotation of full-length DNA sequences

Functional annotations for the nrFLcDNAs were provided according to sequence similarity with public sequence datasets. To estimate the similarity in a protein sequence, nrFLcDNA sequences were queried against protein datasets from NCBI nr, UniProt http://www.uniprot.org/, Arabidopsis protein sequences (TAIR9, http://www.arabidopsis.org/), and rice protein sequences (RAP-DB, http://rapdb.dna.affrc.go.jp/) using the BLASTX and BLASTP. To estimate the similarity to tomato unigenes, nrFLcDNA sequences were queried against the unigene/TC datasets from SGN unigene sequences (draft_Solanum_lycopersicum_transcript_assembly_2009_08_05.fasta, ftp://ftp.sgn.cornell.edu/unigene_builds) and the DFCI Tomato Gene Index (release 12.0, http://compbio.dfci.harvard.edu/tgi/) using the BLASTN. Searches for the protein domains in the amino acid sequences predicted from six open reading frames were performed using InterProScan [42]. Gene ontology (GO) annotations [43] for Arabidopsis genes with the highest similarity to nrFLcDNAs were retrieved from a TAIR GO annotation search http://www.arabidopsis.org/tools/bulk/go/index.jsp. GO annotation documents for InterProScan domains including "interpro2go," "entry.list," and "ParentChildTreeFile.txt" were obtained at the InterPro public ftp site ftp://ftp.ebi.ac.uk/pub/databases/interpro/ in May 2009. Data files of Arabidopsis transcription factors (file named AtTFDB.zip) were downloaded from AGRIS AtTFDB http://arabidopsis.med.ohio-state.edu/AtTFDB/. The difference in the frequency of GO terms was tested by Fisher's exact probability test (P < 0.01).

### Pathway annotations

The SGN tomato unigene (Tomato_200607_build_1, downloaded from SGN ftp site ftp://ftp.sgn.cornell.edu/unigene_builds/) with the highest similarity to each nrFLcDNAs was searched for using BLASTN, and then SGN tomato unigenes that matched nrFLcDNAs at the threshold E-value of < 1e-180 were assigned to tomato metabolic pathways according to LycoCyc version 1.0.1.1 ftp://ftp.sgn.cornell.edu/pathways/lycocyc.dump.txt. Pathway hierarchy was retrieved on May 1, 2009 from "Hierarchical pathway ranking for *Solanum lycopersicum*" http://solcyc.sgn.cornell.edu/LYCO/hierarchical.html. The version of LycoCyc used in this study provides pathway annotations to 1532 SGN unigenes. The difference in the frequency of pathway annotation terms was tested by Fisher's exact probability test (P < 0.05).

### Similarity to genes of other plants

The similarity of nrFLcDNAs to genes of other plants was estimated using tBLASTN with E-value cut-off of 1e-10 against DFCI Gene Indeices of *Hordeum vulgare *(barley), *Triticum aestivum *(wheat), *Zea mays *(maize), *Pinus *(pine), *Picea *(spruce), *Populus *(poplar), *Lotus japonicus*, *Medicago truncatula*, *Glycine max *(soybean), *Citrus sinensis *(orange), *Malus × domestica *(apple), *Vitis vinifera *(grape), *Nicotiana tabacum *(tobacco), and *Solanum tuberosum *(potato) (see Additional file 5: Datasets used for comparison with other plants). GO annotations for DFCI tomato TCs ("LGIGO.071608") were downloaded from the DFCI public ftp site. The difference in the frequency of GO terms was tested by Fisher's exact probability test (P < 0.05).

### Mapping full-length cDNA sequences to tomato genome sequence

Poly(A) tails of the nrFLcDNA sequences were excised, and this set of nrFLcDNAs without poly(A) tails was used for similarity searches against the Prerelease of Tomato Genome Shotgun Sequence (S_lycopersicum_scaffolds_20091201.fa, ftp://ftp.solgenomics.net/tomato_genome/wgs/assembly). Tomato genome scaffold sequences with the highest similarity to a given nrFLcDNA sequence were identified using BLASTN (threshold E-value, 1e-50). Each pair of scaffold and nrFLcDNA sequences was then submitted to exon and intron prediction using the est2genome program [41] by setting scaffold and nrFLcDNA sequences as "genome" and "est," respectively. To analyze numbers and lengths of exons and introns, cut-off value for the alignment length was set as 90% of nrFLcDNA length. Cut-off value for nucleotide identity was set as 90%. To estimate the single nucleotide polymorphisms (SNPs) between nrFLcDNA and scaffold sequences, the number of nucleotide mismatches was counted in the nrFLcDNA-scaffold pairs having 'identity' in BLAST output equal to or larger than 99.5%.

### Database

The data presented in this study, including full-length cDNA sequences of the 13,227 HTCs, the results of similarity searches against nr, UniProt, TAIR, RAP-DB, SGN tomato unigenes, DFCI tomato TCs, and InterProScan, and mapping of full-length sequences against SGN tomato BAC sequences are available at the tomato full-length cDNA database KaFTom http://www.pgb.kazusa.or.jp/kaftom/. KaFTom is accessible directly or via the National Bioresource Project Tomato website http://tomato.nbrp.jp.

## Abbreviations

(HTC): high throughput cDNA sequence; (nrFLcDNA): non-redundant full-length cDNA; (CDS): coding sequence; (UTR): untranslated region; (DFCI): Dana-Farber Cancer Institute; (SGN): SOL genomics network; (TC): tentative consensus.

## Authors' contributions

This study was conceived and directed by KA and DS. RNA samples were prepared by NS, KS, TT, MW, HT, YW, ME, MK, YI, MK, SF, AO, TA, YS, KY, SS, TO, and HE. cDNA libraries were constructed by KA, SS, HE, and DS. Sequencing was carried out by KS, AK, TS, TT, MW, MT, TN, TS, and YK. Assembly of the full-length sequences was performed by KS, AK, TS, and MW under the direction of KY. Sequence analyses and other bioinformatics were directed by KY, and performed by AS, SK, NY, and KA. Database construction was directed by KY and performed by AS, SK, and KO. KA wrote the paper. All authors approved the final manuscript.

## Supplementary Material

Additional file 1 **Tom tissues used for RNA preparation**. Detailed description of Micro-Tom tissues used for RNA preparation including tissue positions, treatments, and age of plants when tissues were harvested. Click here for file

Additional file 2 **Clone number list**. Lists of clone numbers of nrFLcDNAs-sets appeared in the text Click here for file

Additional file 3 **cDNA deived from non-coding RNA**. Clone numbers and annotations of nrFLcDNAs that matched to non-coding RNAs registered in NONCODE database. Click here for file

Additional file 4 **Predicted CDS of 11,597 non-redundant HTCs**. A list of amino acid sequences predicted for 11,597 non-redundant HTCs and results of BLASTP search against protein datasets of nr and tomato SBM database http://www.kazusa.or.jp/tomato/. Click here for file

Additional file 5 **Datasets used for comparative analysis with other plants**. Name and version of protein- and tentative consensus-datasets used for comparison of nrFLcDNAs with gene of other plants. Click here for file
