# Supplementary material for: Large-scale analysis of full-length cDNAs from the tomato (Solanum lycopersicum) cultivar Micro-Tom, a reference system for the Solanaceae genomics
Source: BMC Genomics. 2010 Mar 30;11:210. doi: 10.1186/1471-2164-11-210 (PMC2859864; doi:10.1186/1471-2164-11-210)
Supplement: Additional file 1 — Tom tissues used for RNA preparation. Detailed description of Micro-Tom tissues used for RNA preparation including tissue positions, treatments, and age of plants when tissues were harvested. [file 1471-2164-11-210-S1.DOC]

**Additional file 1.** Micro-Tom tissues used for RNA preparation.

|  | Organ | Treatment | Tissue | Condition | Library |
| --- | --- | --- | --- | --- | --- |
| 1 | Leaf | CMV(strain TN) and satellite RNA | Inoculated leaves | 16 dpi (21-day old) | LEFL1 |
| 2 |  | CMV(strain TN) and satellite RNA | Systemic upper leaves | 16 dpi (21-day old) | LEFL1 |
| 3 |  | ToMV (strain Lta1) | Inoculated leaves | 7 dpi (28-day old) | LEFL1 |
| 4 |  | ToMV (strain Lta1) | Inoculated leaves | 14 dpi (35-day old) | LEFL1 |
| 5 |  | ToMV (strain Lta1) | Systemic upper leaves | 14 dpi (35-day old) | LEFL1 |
| 6 |  | ToMV (strain L_11_A) | Inoculated leaves | 7 dpi (28-day old) | LEFL1 |
| 7 |  | ToMV (strain L_11_A) | Inoculated leaves | 14 dpi (35-day old) | LEFL1 |
| 8 |  | ToMV (strain L_11_A) | Systemic upper leaves | 14 dpi (35-day old) | LEFL1 |
| 9 |  | ToMV (strain L2a) | Inoculated leaves | 7 dpi (28-day old) | LEFL1 |
| 10 |  | ToMV (strain L2a) | Inoculated leaves | 14 dpi (35-day old) | LEFL1 |
| 11 |  | ToMV (strain L2a) | Systemic upper leaves | 14 dpi (35-day old) | LEFL1 |
| 12 |  | ToMV (strain Ltb1) | Inoculated leaves | 7 dpi (28-day old) | LEFL1 |
| 13 |  | ToMV (strain Ltb1) | Inoculated leaves | 14 dpi (35-day old) | LEFL1 |
| 14 |  | ToMV (strain Ltb1) | Systemic upper leaves | 14 dpi (35-day old) | LEFL1 |
| 15 |  | ToMV (strain LJB) | Inoculated leaves | 7 dpi (28-day old) | LEFL1 |
| 16 |  | ToMV (strain LJB) | Inoculated leaves | 14 dpi (35-day old) | LEFL1 |
| 17 |  | ToMV (strain LJB) | Systemic upper leaves | 14 dpi (35-day old) | LEFL1 |
| 18 |  | Non-pathogenic *Alternaria alternata* | Inoculated leaves | 6 hpi (5-week old) | LEFL1 |
| 19 |  | Non-pathogenic *Alternaria alternata* | Inoculated leaves | 24 hpi (5-week old) | LEFL1 |
| 20 |  | *Alternaria alternate* f. sp. *lycopersici* (As-27) | Inoculated leaves | 6 hpi (5-week old) | LEFL1 |
| 21 |  | *Alternaria alternate* f. sp. *lycopersici* (As-27) | Inoculated leaves | 24 hpi (5-week old) | LEFL1 |
| 22 |  | *Corynespora cassiicola* (strain C95001) | Inoculated leaves | 6 hpi (3-week old) | LEFL1 |
| 23 |  | *Corynespora cassiicola* (strain C95001) | Inoculated leaves | 24 hpi (3-week old) | LEFL1 |
| 24 |  | *Corynespora cassiicola* (strain LC93020) | Inoculated leaves | 6 hpi (3-week old) | LEFL1 |
| 25 |  | *Corynespora cassiicola* (strain LC93020) | Inoculated leaves | 24 hpi (3-week old) | LEFL1 |
| 26 |  | *Cladosporium fulvum* (strain 210 race 2, 4, and 11) | Inoculated leaves | 5 dpi (40-day old) | LEFL1 |
| 27 |  | *Cladosporium fulvum* (strain 211 race 2) | Inoculated leaves | 5 dpi (40-day old) | LEFL1 |
| 28 |  | *Cladosporium fulvum* (strain 217 race 0) | Inoculated leaves | 5 dpi (40-day old) | LEFL1 |
| 29 |  | *Fusarium oxysporum* f. sp. *lycopersici* race 1 | Leaves | Inoculated to root,  7 dpi (4-week old) | LEFL1 |
| 30 |  | *Fusarium oxysporum* f. sp. *lycopersici* race 2 | Leaves | Inoculated to root,  7 dpi (4-week old) | LEFL1 |
| 31 |  | Non-pathogenic *Fusarium oxysporum* | Leaves | Inoculated to root,  7 dpi (4-week old) | LEFL1 |
| 32 |  | *Pseudomonas syringae* pv. *tomato* DC3000 | Spray-inoculated leaves  (2 x 10^8^ cfu/ml) | 24 hpi (6-week old) | LEFL1 |
| 33 |  | *Pseudomonas syringae* pv. *tabaci* isolate 6605, wild type | Spray-inoculated leaves  (2 x 10^8^ cfu/ml) | 24 hpi (6-week old) | LEFL1 |
| 34 |  | *Pseudomonas syringae* pv. *tabaci* isolate 6605, *ΔfliC* | Spray-inoculated leaves  (2 x 10^8^ cfu/ml) | 24 hpi (6-week old) | LEFL1 |
| 35 |  | *Pseudomonas syringae* pv. *tabaci* isolate 6605, *ΔfliD* | Spray-inoculated leaves  (2 x 10^8^ cfu/ml) | 24 hpi (6-week old) | LEFL1 |
| 36 |  | Flagellin from *Pseudomonas syringae* pv. *tabaci* isolate 6605 (100 µg/ml) | Sprayed leaves | 3 hps (6-week old) | LEFL1 |
| 37 |  | flg22 (3.2 µM) | Sprayed leaves | 3 hps (6-week old) | LEFL1 |
| 38 |  | Probenazole (100 μg/ml) | Sprayed leaves | 4 dps (4-week old) | LEFL1 |
| 39 |  | Validamycin A (100 μg/ml) | Sprayed leaves | 4 dps (4-week old) | LEFL1 |
| 40 |  | Acibenzolar-S-methyl (100 μg/ml) | Sprayed leaves | 4 dps (4-week old) | LEFL1 |
| 41 |  | Validoxylamine A (100 μg/ml) | Sprayed leaves | 4 dps (4-week old) | LEFL1 |
| 42 |  | Salicilic acid (0.5 mM) | Sprayed leaves | 2 dps (4-week old) | LEFL1 |
| 43 |  | Methyl jasmonic acid (50μM) | Sprayed leaves | 2 dps (4-week old) | LEFL1 |
| 44 | Fruit | Mature green | Pericarp | 40 daa, year 2003 | FC |
| 45 |  | Breaker | Pericarp | 42 daa, year 2003 | FC |
| 46 |  | Turning | Pericarp | 48 daa, year 2003 | FC |
| 47 |  | Red ripe | Pericarp | 50 daa, year 2003 | FC |
| 48 |  | Mature green | Pericarp | 40 daa, year 2004 | LEFL2 |
| 49 |  | Breaker | Pericarp | 42 daa, year 2004 | LEFL2 |
| 50 |  | Turning | Pericarp | 48 daa, year 2004 | LEFL2 |
| 51 |  | Red ripe | Pericarp | 50 daa, year 2004 | LEFL2 |
| 52 | Root | Roots from plants without flower | Roots | 6-week old | LEFL3 |
| 53 |  | Roots from plants without flower | Roots | 10-week old | LEFL3 |
| 54 |  | Roots from flowered plants | Roots | 10- week old | LEFL3 |
| 55 |  | Roots from flowered plants | Roots | 12- week old | LEFL3 |
| 56 |  | *Fusarium oxysporum* race 2 | Roots | 1 dpi, 4-week old | LEFL3 |
| 57 |  | *Fusarium oxysporum* race 2 | Roots | 7 dpi, 5-week old | LEFL3 |
| 58 |  | *Fusarium oxysporum* race 2 | Roots | 14 dpi, 6-week old | LEFL3 |
| 59 |  | *Fusarium oxysporum* race 2 | Roots | 21 dpi, 7-week old | LEFL3 |
| 60 |  | *Fusarium oxysporum* race 2 | Roots | 1 dpi, 8-week old | LEFL3 |
| 61 |  | *Fusarium oxysporum* race 2 | Roots | 7 dpi, 9-week old | LEFL3 |
| 62 |  | *Fusarium oxysporum* race 2 | Roots | 14 dpi, 10-week old | LEFL3 |
| 63 |  | *Fusarium oxysporum* race 2 | Roots | 21 dpi, 11-week old | LEFL3 |
