# Supplementary material for: Large-scale analysis of full-length cDNAs from the tomato (Solanum lycopersicum) cultivar Micro-Tom, a reference system for the Solanaceae genomics
Source: BMC Genomics. 2010 Mar 30;11:210. doi: 10.1186/1471-2164-11-210 (PMC2859864; doi:10.1186/1471-2164-11-210)
Supplement: Additional file 3 — cDNA deived from non-coding RNA. Clone numbers and annotations of nrFLcDNAs that matched to non-coding RNAs registered in NONCODE database. [file 1471-2164-11-210-S3.DOC]

**Additional file 3.** cDNAs derived from non-coding RNA.

| nrFLcDNA | Length (bp) | ncRNA | Length of ncRNA (nt) | ncRNA class  (process function class) |
| --- | --- | --- | --- | --- |
| FC25DB10 | 563 | n5000_u4401_U34808 | 474 | TPSI1 RNA^a^ (Miscfunction mRNAlike) |
| LEFL3003O13 | 489 | n5000_u4401_U34808 | 474 | TPSI1 RNA^a^ (Miscfunction mRNAlike) |
| LEFL3054J22 | 553 | n202769_u198041_AK  016310 | 541 | mRNAlike RNA (Miscfunction mRNAlike) |

^a^Tomato phosphate starvation-induced gene (TPSI1).
