# Supplementary material for: Large-scale analysis of full-length cDNAs from the tomato (Solanum lycopersicum) cultivar Micro-Tom, a reference system for the Solanaceae genomics
Source: BMC Genomics. 2010 Mar 30;11:210. doi: 10.1186/1471-2164-11-210 (PMC2859864; doi:10.1186/1471-2164-11-210)
Supplement: Additional file 5 — Datasets used for comparative analysis with other plants. Name and version of protein- and tentative consensus-datasets used for comparison of nrFLcDNAs with gene of other plants. [file 1471-2164-11-210-S5.DOC]

**Additional file 5.** Datasets used for comparative analysis with other plants.

| Dataset | Source |
| --- | --- |
| *Arabidopsis thaliana* protein | TAIR9_pep_20090619 |
| *Oryza sativa* protein | RAP-DB orf_amino_withgff (2006/08/28) |
| *Hordeum vulgare* (barley) transcript | DFCI Barley Gene Index release10.0 (2008/06/26) |
| *Triticum aestivum* (wheat) transcript | DFCI Wheat Gene Index release11.0 (2008/07/15) |
| *Zea mays* (maize) transcript | DFCI Maize Gene Index release19.0 (2009/05/29) |
| *Pinus* (pine) transcript | DFCI Pine Gene Index release7.0 (2008/07/23) |
| *Picea* (spruce) transcript | DFCI Spruce Gene Index release3.0 (2008/07/15) |
| *Populus* (poplar) transcript | DFCI Poplar Gene Index release4.0 (2008/07/24) |
| *Lotus japonicus* transcript | DFCI L.japonicus Gene Index release5.0 (2009/05/29) |
| *Medicago truncatula* transcript | DFCI Medicago Gene Index release9.0 (2008/07/17) |
| *Glycine max* (soybean) transcript | DFCI Soybean Gene Index release14.0 (2009/05/29) |
| *Citrus sinensis* (orange) transcript | DFCI Orange Gene Index release1.0 (2008/07/01) |
| *Malus x domestica* (apple) transcript | DFCI Apple Gene Index release2.0 (2009/05/29) |
| *Vitis vinifera* (grape) transcript | DFCI Grape Gene Index release6.0 (2008/07/30) |
| *Nicotiana tabacum* (tobacco) transcript | DFCI Tobacco Gene Index release5.0 (2009/05/29) |
| *Solanum tuberosum* (potato) transcript | DFCI Potato Gene Index release12.0 (2008/07/24) |
| *Solanum lycopersicum* (tomato) transcript | DFCI Tomato Gene Index release12.0 (2008/07/16) |
| *Solanum lycopersicum* (tomato) transcript | SGN Tomato_20090805 (2009/08/05) |
